# Three new species of saddled loricariid catfishes, and a review of *Hemiancistrus*, *Peckoltia*, and allied genera (Siluriformes)

**DOI:** 10.3897/zookeys.480.6540

**Published:** 2015-02-02

**Authors:** Jonathan W. Armbruster, David C. Werneke, Milton Tan

**Affiliations:** 1 Department of Biological Sciences, 101 Life Sciences Building, Auburn University, AL 36849, USA

**Keywords:** Ancistrini, Hypostominae, *Peckoltia*, Siluriformes, Systematics, Taxonomy

## Abstract

Three new species of saddled hypostomine loricariids are described. According to a recent phylogenetic analysis, these species are members of the genus *Peckoltia*. The species differ from all described *Peckoltia* except *Peckoltia furcata* and *Peckoltia sabaji* by having the dentaries meet at an angle greater than 90°. The species also have similarities to *Hemiancistrus*, and can be separated from all described species by having dorsal saddles. We discuss the taxonomy of *Peckoltia*, *Hemiancistrus*, and allied genera and recognize *Ancistomus* as valid for *Peckoltia feldbergae*, *Hemiancistrus micrommatos*, *Ancistrus snethlageae*, *Hemiancistrus spilomma*, and *Hemiancistrus spinosissimus*. We recommend descriptions of genera for several clades of *Hemiancistrus* and restriction of *Hemiancistrus* to the type species of the genus, *Hemiancistrus medians*. *Chaetostomus macrops* is transferred to *Pseudancistrus* and recognized as a junior synonym of *Pseudancistrus megacephalus*. The *Hemiancistrus annectens* group of species (*Hemiancistrus annectens*, *Hemiancistrus argus*, *Hypostomus aspidolepis*, *Hemiancistrus fugleri*, *Hemiancistrus holostictus*, *Hemiancistrus maracaiboensis*, *Hemiancistrus panamensis*, *Hemiancistrus wilsoni*) are recognized in *Hypostomus*. Multivariate analysis reveals that the newly described species differ from one another in shape space, but overlap broadly with other *Peckoltia* (*Peckoltia lujani*), narrowly with other *Peckoltia* (*Peckoltia greedoi*), or broadly with *Etsaputu* (*Peckoltia ephippiata*).

## Introduction

The Loricariidae, or suckermouth armored catfishes, comprise over 800 species and present numerous difficult taxonomic problems. Among the worst problems is the identity of *Peckoltia* Miranda Ribeiro and allied genera like *Ancistomus* Isbrücker and Seidel and *Hemiancistrus* Bleeker. The genus *Peckoltia* was described by Miranda Ribeiro (1912) with *Chaetostomus vittatus* (Steindachner, 1881) as the type. The definition has since remained unclear. Armbruster (2008) revised *Peckoltia* *sensu stricto*, and he defined the genus as having dentaries meeting at an angle less than 90°, but lacking any of the synapomorphies of other ancistrin genera with angled jaws. All other ancistrin species without characteristics that united them into other genera were lumped into *Hemiancistrus* by Armbruster (2008). Despite this definition, other researchers used an expanded definition of *Peckoltia* without any clear delineations, including such species as *Ancistrus snethlageae* and *Peckoltia sabaji*, which Armbruster (2008) considered part of the polyphyletic *Hemiancistrus* (Oliveira et al. 2010, 2012; Fisch-Muller et al. 2012). The result was a likely polyphyletic *Peckoltia* in addition to a polyphyletic *Hemiancistrus*. This confusion on the identity of the species is due to a lack of clear morphological groups into which species can be assigned, and attempts to constrain species into two artificial taxa. Because of the lack of a clear morphological signal, a molecular phylogeny was needed to make some sense of the taxa.

Lujan et al. (2015) provide the largest phylogenetic analysis of loricariids to date focusing on the Hypostominae. The analysis included 181 species and 91 genera of loricariids, and was based on two mitochondrial and three nuclear genes. The study offers a compelling answer to why the taxa are so complex: morphology of loricariids is incredibly plastic, with lots of convergence. Lujan et al. (2015) showed that the genera *Peckoltia* and *Hemiancistrus* are not monophyletic as previously defined, and offer a new taxonomy for species of *Peckoltia* and *Hemiancistrus* (expanded upon here in Table 1). Although morphological support for most of the clades in the molecular phylogeny is currently unknown, the phylogeny offers a framework for describing new species of loricariids, and no morphological support was present for any of the previous taxonomic hypotheses. Lujan et al. (2015) suggested that *Peckoltia sabaji* does belong in *Peckoltia* as well as the three species described herein; however, Lujan et al. (2015) also suggested that *Ancistomus* is a valid genus, and it includes *Peckoltia feldbergae*. *Chaetostomus bachi* was included in *Peckoltia* by Armbruster (2008), but the unusual species was not closely related to the remainder of *Peckoltia* in the molecular phylogeny, and *Peckoltichthys* was resurrected as monotypic. *Hemiancistrus* likely only contains *Hemiancistrus medians*, and several species groups within *Hemiancistrus* require new genera (Table 1). *Etsaputu* was found nested within *Peckoltia*, but Lujan et al. do not sink the genus as it has a very unusual anatomy (lacks a fully evertible cheek apparatus), and the placement of the genus needs further work.

**Table 1.** Species once part of *Hemiancistrus* and/or *Peckoltia* and their current taxonomy. Current names are per this study, Isbrücker 1980, 2001; Fisch-Muller 2003; Weber 2003; Armbruster 2004, 2005, 2008; or original descriptions.

Original name	Current name	Author	Notes
*Ancistrus annectens*	*Hypostomus annectens*	Regan, 1904	comb. n.
*Ancistrus brachyurus*	*Dekeyseria brachyura*	Kner, 1854	
*Ancistrus medians*	*Hemiancistrus medians*	Kner, 1854	
*Ancistrus multispinis*	*Peckoltia multispinis*	Holly, 1929	
*Ancistrus pulcher*	*Dekeyseria pulchra*	Steindachner, 1915	
*Ancistrus salgadae*	*Hypostomus salgadae*	Fowler, 1941	comb. n.
*Ancistrus scaphirhynchus*	*Dekeyseria scaphirhyncha*	Kner, 1854	
*Ancistrus snethlageae*	*Ancistomus snethlageae*	Steindachner, 1911	
*Ancistrus yaravi*	*Neblinichthys yaravi*	Steindachner, 1915	
*Chaetostomus macrops*	‘Pseudancistrus’ megacephalus	Lütken, 1874	
*Chaetostomus aspidolepis*	*Hypostomus aspidolepis*	Günther, 1867	comb. n.
*Chaetostomus bachi*	*Peckoltichthys bachi*	Boulenger, 1898	
*Chaetostomus furcatus*	*Peckoltia furcata*	Fowler, 1940	
*Chaetostomus megacephalus*	‘Pseudancistrus’ megacephalus	Günther, 1868	
*Chaetostomus oligospilus*	*Peckoltia oligospila*	Günther, 1864	
*Chaetostomus platycephalus*	*Cordylancistrus platycephalus*	Boulenger, 1898	
*Chaetostomus vittatus*	*Peckoltia vittata*	Steindachner, 1881	
*Hemiancistrus albocinctus*	*Ancistrus multispinis*	Ahl, 1936	
*Hemiancistrus arenarius*	*Peckoltichthys bachi*	Eigenmann & Allen, 1942	comb. n.
*Hemiancistrus braueri*	*Peckoltia braueri*	Eigenmann, 1912	
*Hemiancistrus brevis*	*Peckoltia brevis*	La Monte, 1935	
*Hemiancistrus caquetae*	*Lasiancistrus schomburgkii*	Fowler, 1945	
*Hemiancistrus castelnaui*	*Lasiancistrus schomburgkii*	Miranda Ribeiro, 1911	
*Hemiancistrus cerrado*	‘Hemiancistrus’ cerrado	de Souza et al., 2008	‘Hemiancistrus’ chlorostictus group
*Hemiancistrus chlorostictus*	‘Hemiancistrus’ chlorostictus	Cardoso & Malabarba, 1999	‘Hemiancistrus’ chlorostictus group
*Hemiancistrus daguae*	*Cordylancistrus daguae*	Eigenmann, 1912	
*Hemiancistrus fugleri*	*Hypostomus annectens*	Ovchynnyk, 1971	syn. n.
*Hemiancistrus fuliginosus*	‘Hemiancistrus’ fuliginosus	Cardoso & Malabarba, 1999	‘Hemiancistrus’ chlorostictus group
*Hemiancistrus guahiborum*	‘Hemiancistrus’ guahiborum	Werneke et al., 2005	‘Hemiancistrus’ guahibroum group
*Hemiancistrus hammarlundi*	‘Hemiancistrus’ landoni	Rendahl, 1937	syn. n.
*Hemiancistrus holostictus*	‘Hypostomus’ holostictus	Regan, 1913	comb. n.
*Hemiancistrus landoni*	‘Hemiancistrus’ landoni	Eigenmann, 1916	‘Hemiancistrus’ landoni group
*Hemiancistrus longipinnis*	*Baryancistrus longipinnis*	Kindle, 1895	
*Hemiancistrus maracaiboensis*	*Hypostomus maracaiboensis*	Schultz, 1944	comb. n.
*Hemiancistrus mayoloi*	*Lasiancistrus caucanus*	Eigenmann, 1912	
*Hemiancistrus megalopteryx*	‘Hemiancistrus’ megalopteryx	Cardoso, 2004	‘Hemiancistrus’ chlorostictus group
*Hemiancistrus meizospilos*	‘Hemiancistrus’ meizospilos	Cardoso & da Silva, 2004	‘Hemiancistrus’ chlorostictus group
*Hemiancistrus micrommatos*	*Ancistomus micrommatos*	Cardoso & Lucinda, 2003	comb. n.
*Hemiancistrus niceforoi*	*Hypostomus niceforoi*	Fowler, 1943	
*Hemiancistrus niger*	*Guyanancistrus niger*	Norman, 1926	
*Hemiancistrus pankimpuju*	*Peckoltia pankimpuju*	Lujan & Chamon, 2008	comb. n.
*Hemiancistrus platyrhynchus*	*Chaetostoma platyrhyncha*	Fowler, 1943	
*Hemiancistrus punctulatus*	‘Hemiancistrus’ punctulatus	Cardoso & Malabarba, 1999	‘Hemiancistrus’ chlorostictus group
*Hemiancistrus spilomma*	*Ancistomus spilomma*	Cardoso & Lucinda, 2003	comb. n.
*Hemiancistrus spinosissimus*	*Ancistomus spinosissimus*	Cardoso & Lucinda, 2003	comb. n.
*Hemiancistrus subviridis*	‘Hemiancistrus’ subviridis	Werneke et al., 2005	‘Hemiancistrus’ guahibroum group
*Hemiancistrus ucayalensis*	*Peckoltichthys bachi*	Fowler, 1940	comb. n.
*Hemiancistrus votouro*	‘Hemiancistrus’ votouro	Cardoso and da Silva, 2004	‘Hemiancistrus’ chlorostictus group
*Hemiancistrus wilsoni*	*Hypostomus wilsoni*	Eigenmann, 1918	comb. n.
*Hypostomus itacua*	*Hypostomus itacua* *incertae sedis*	Valenciennes, 1836	probably is a *Hypostomus*
*Hypostomus pictus*	*Lasiancistrus schomburgkii*	Castelnau, 1855	
*Peckoltia caenosa*	*Peckoltia caenosa*	Armbruster, 2008	
*Peckoltia capitulata*	*Peckoltia capitulata*	Fisch-Muller & Covain, 2012	
*Peckoltia cavatica*	*Peckoltia cavatica*	Armbruster & Werneke, 2005	
*Peckoltia compta*	*Peckoltia compta*	de Oliveira et al., 2010	
*Peckoltia feldbergae*	*Ancistomus feldbergae*	de Oliveira et al., 2012	comb. n.
*Peckoltia lineola*	*Peckoltia lineola*	Armbruster, 2008	
*Peckoltia otali*	*Peckoltia otali*	Fisch-Muller & Covain, 2012	
*Peckoltia sabaji*	*Peckoltia sabaji*	Armbruster, 2003	
*Peckoltia simulata*	*Peckoltia simulata*	Fisch-Muller & Covain, 2012	
*Peckoltichthys filicaudatus*	*Peckoltichthys bachi*	Miranda Ribeiro, 1917	
*Peckoltichthys kuhlmanni*	*Peckoltia vittata*	Miranda Ribeiro, 1920	
*Plecostomus niveatus*	*Dekeyseria niveata*	La Monte, 1929	
*Plecostomus panamensis panamensis*	*Hypostomus aspidolepis*	Eigenmann, 1922	

In this paper, we describe three new species of *Peckoltia*. These species differ from *Peckoltia* *sensu stricto*. by having their jaws meet at an angle greater than 90°, but share with most species of *Peckoltia* the presence of dorsal saddles (absent in *Hemiancistrus* *sensu lato*). In addition, we discuss the implications of the molecular phylogeny of Lujan et al. (2015) on the taxonomy of *Peckoltia*, *Hemiancistrus*, and allied genera, and we recognize several species groups that may represent new genera.

## Methods

Methods follow Armbruster (2003) with the addition of counts of mid-dorsal and mid-ventral plates (the number of plates in these series from the head to caudal fin and excluding the last plate, which is beyond the hypural). Institutional abbreviations are as in Sabaj Pérez (2014). Names of skeletal characteristics are as in Schaefer (1987) and of plate rows as in Schaefer (1997). A principal component analysis for the morphometric data of all *Peckoltia* examined in Armbruster (2008) and the specimens below was performed using a covariate matrix and log-transformed measurements in JMP (Vers. 5.01a, SAS Institute, 2002). We have not examined specimens of recently described species (*Peckoltia capitulata*, *Peckoltia compta*, *Peckoltia otali*, and *Peckoltia stimulata*). Principal component one was excluded from further analysis because it represented overall size differences (vs. relative size differences). The following abbreviations are used in the text: dr. = drainage, nm. = not measured. Full morphometric dataset is presented in Suppl. material 1, locality information for species described below is presented in Suppl. material 2. Character state data for *Hemiancistrus medians* based on Armbruster (2004a, 2008) in Suppl. material 3.

## Results

### Morphometrics

**Figure 1.**
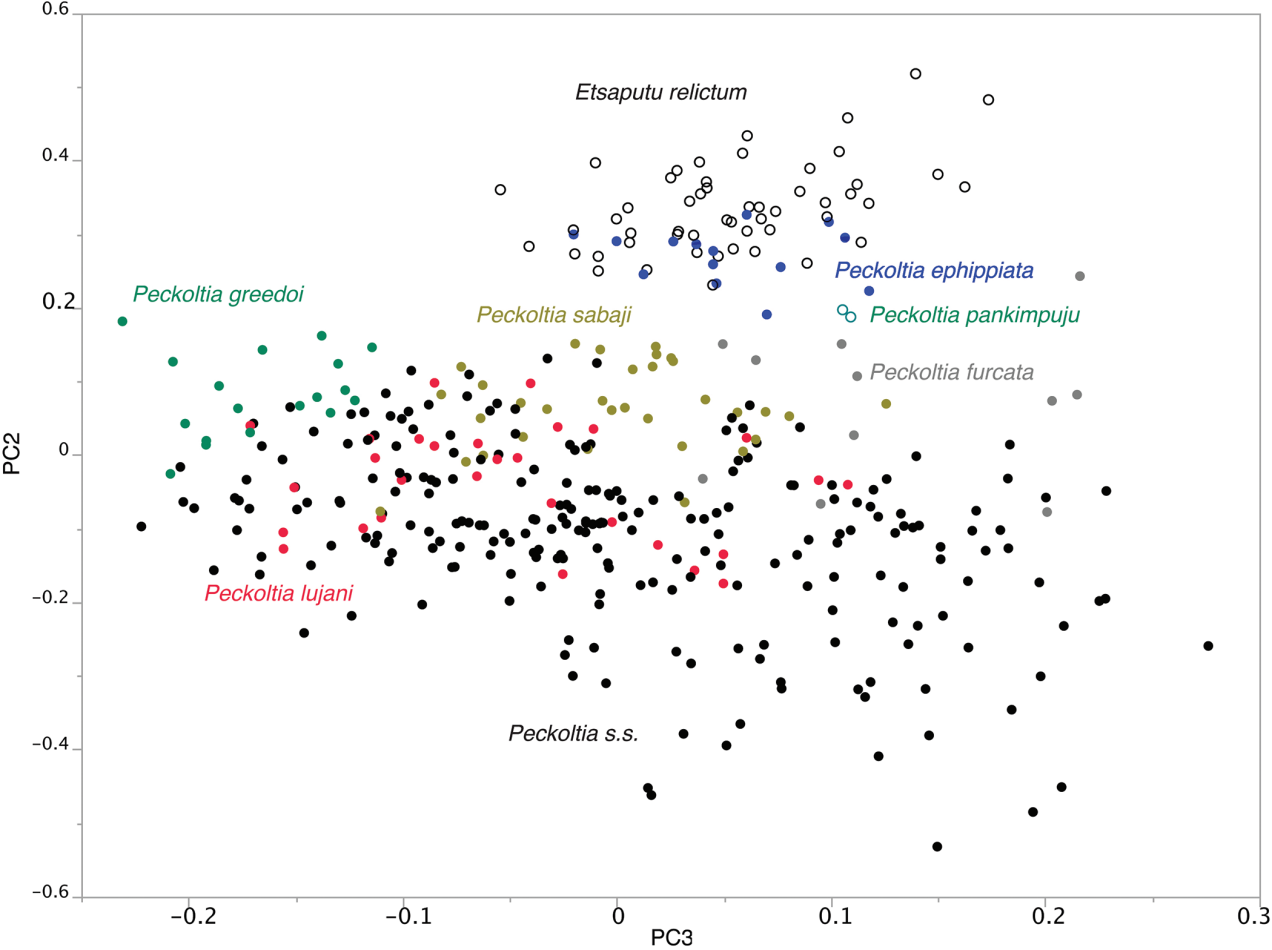
Principal Component Analysis of species of *Peckoltia* with *Peckoltia* *s.s.* being those *Peckoltia* with jaws forming an angle less than 90° (some *Peckoltia furcata* also have this low jaw angle). PC2 is most strongly influenced negatively by barbel length, internares with, and caudal-peduncle depth, and positively by dentary length, premaxillary length, and mouth width. PC3 is most strongly influenced negatively by barbel length, premaxillary length, and dorsal-adipose distance, and positively by adipose-upper caudal distance, adipose spine length, and head-dorsal distance.

The three new species show little overlap with one another in the PCA (Fig. 1). *Peckoltia lujani* overlaps *Peckoltia* *sensu stricto* (*Peckoltia* *sensu* Armbruster, 2008, minus *Peckoltichthys bachi*), *Peckoltia sabaji*, and *Peckoltia furcata*. *Peckoltia greedoi* overlaps *Peckoltia* *s.s.* and *Peckoltia lujani* slightly. *Peckoltia ephippiata* overlaps only *Etsaputu relictum*.

### Peckoltia ephippiata

Taxon classificationAnimaliaSiluriformesLoricariidae

Armbruster, Werneke & Tan sp. n.

http://zoobank.org/2BA0FAC3-FCA5-404D-BA5E-C2232FCD636F

Fig. 2 , Table 2

#### Type locality.

Brazil, South America

#### Holotype.

MCP 35627, 1, 101.7 mm SL, BRAZIL, Rôndonia, Presidente Médici. rio Madeira dr., rio Leitão on highway BR-364, about 5 km N of Presidente Médici, -11.1328°, -061.9008°, 15 Jul 2004, R.E. Reis, P.C. Lehmann, F.C. Lima, and E.H.L. Pereira.

#### Paratypes.

ANSP 197614, 2, 60.4–92.5, AUM 65116, 2, 64.1–96.4, MCP 48395, 13, 48.2–97.7, MNRJ 42662, 2, 66.0–89.7, UF 237091, 2, 55.6–82.6, same locality data as holotype.

#### Diagnosis.

*Peckoltia ephippiata* can be separated from *Peckoltia pankimpuju* by having well developed color and eyes; from all other *Peckoltia* by having no spots or bands in the dorsal fin; from all except *Peckoltia greedoi* by having small, very faint spots on the head (vs. large spots, mottling, short lines, or thick dark areas, always much more intense than the weak spots in *Peckoltia ephippiata*; *Peckoltia greedoi* has a uniformly dark head, but the small faint spots of *Peckoltia ephippiata* can appear uniformly dark without closer inspection); from all *Peckoltia* except *Peckoltia furcata*, *Peckoltia greedoi*, *Peckoltia lujani*, *Peckoltia pankimpuju*, and *Peckoltia sabaji* by having the dentaries meet at an angle greater than 90°; from *Peckoltia greedoi* and *Peckoltia lujani* by lacking bands in the dorsal fin, rays light and membranes dark (vs. bands present), by having more teeth (*Peckoltia ephippiata*: 39–72 dentary, 41–73 premaxillary; *Peckoltia greedoi*: 16–39 dentary, 20–38 premaxillary; *Peckoltia lujani*: 20–37 dentary, 23–45 premaxillary), by having slight keels on the lateral plates, particularly the median series (vs. keels absent), and by having platelets on the central region of the abdomen posterior to the pectoral girdle present (vs. platelets maximally present below pectoral girdle and in a narrow, lateral column just posterior to pectoral fin, and below pelvic girdle); and from *Peckoltia lujani* by having the pectoral-fin spine relaxed position angled dorsally, pointing at insertion of dorsal fin (vs. pectoral-fin spine angled only slightly dorsally, pointing maximally to dorsal insertion of caudal fin) and by the pectoral-fin spine reaching two or more plates of the ventral series beyond the pelvic base when adpressed ventral to pelvic fin (vs. less than one plate).

*Peckoltia ephippiata* differs from *Etsaputu* by having greater than six evertible cheek odontodes, the largest of which extends posterior to the eye (vs. six or fewer, the largest not extending beyond the exposed portion of the opercle). *Peckoltia ephippiata* can be separated from *Hemiancistrus* (except ‘Hemiancistrus’ landoni) and *Ancistomus* by having prominent dorsal saddles (vs. dark or light spots or entirely dark); and from all *Hemiancistrus* and *Ancistomus* by having bands in the caudal fin and no free spots (vs. bands absent or present with some free spots). *Peckoltia ephippiata* can be separated from *Peckoltichthys bachi* by having small, faint spots on the head (vs. large dark spots or mottling); by having the eyes high on the head with the dorsal rim of the orbit higher than the interorbital space (vs. low on the head, dorsal rim of orbit lower than interorbital space), and by having small plates on the abdomen (vs. relatively large).

#### Description.

Morphometrics in Table 2. Counts and measurements based on 18 specimens. Largest specimen examined 101.7 mm SL. Body moderately elongate. Head and nape forming arc from tip of snout to insertion of dorsal fin. Dorsal slope decreasing in straight line to insertion of dorsal procurrent caudal rays then ascending to caudal fin. Body depth greatest below insertion of dorsal fin. Ventral profile flat to caudal fin. Caudal peduncle triangular in cross section with dorsal surface flattened. Body widest at insertion of pectoral fins, narrowest at insertion of caudal fin. Snout rounded.

**Table 2.** Selected morphometrics of *Peckoltia ephippiata*. Numbers in parentheses refer to landmark numbers in Armbruster (2003).

	Holotype	N	Mean	SD	Min	Max
SL, mm (1–20)	101.7	20			48.2	101.7
%SL						
Predorsal Length (1–10)	40.8	20	42.1	1.3	40.4	45.3
Head Length (1–7)	32.1	20	33.8	1.4	31.9	37.3
Head–dorsal Length (7–10)	9.7	20	8.7	0.7	7.1	10.0
Cleithral Width (8–9)	26.9	20	28.4	0.9	26.9	30.2
Head-pectoral Length (1–12)	24.3	20	24.7	1.2	22.9	27.5
Thorax Length (12–13)	22.7	20	23.3	1.1	20.4	25.2
Pectoral-spine Length (12–29)	28.8	20	31.3	1.2	28.3	32.8
Abdominal Length (13–14)	21.7	20	22.3	0.8	21.2	24.0
Pelvic-spine Length (13–30)	25.3	19	25.8	1.8	23.3	30.4
Postanal Length (14–15)	35.8	20	36.0	1.0	34.3	37.8
Anal-fin spine Length (14–31)	15.9	18	14.5	1.4	11.7	16.9
Dorsal–pectoral Distance (10–12)	26.6	20	27.7	1.1	26.1	29.8
Dorsal spine Length (10–11)	32.9	16	32.9	1.4	29.8	34.9
Dorsal-pelvic Distance (10–13)	21.7	20	22.4	1.1	20.3	24.6
Dorsal-fin base Length (10–16)	28.1	20	27.3	1.1	25.2	30.1
Dorsal-adipose Distance (16–17)	14.9	20	14.4	1.4	11.8	16.9
Adipose-spine Length (17–18)	11.4	20	11.6	1.2	8.8	13.1
Adipose-upper caudal Distance (17–19)	16.2	20	17.5	1.1	15.7	19.7
Caudal-peduncle Depth (15–19)	12.2	20	13.0	0.9	11.7	14.8
Adipose-lower caudal Distance (15–17)	24.5	20	25.4	1.6	22.6	28.3
Adipose-anal Distance (14–17)	20.1	20	18.6	1.0	16.2	20.5
Dorsal-anal Distance (14–16)	15.1	20	14.9	0.4	14.0	15.7
Pelvic-dorsal Distance (13–16)	25.1	20	24.9	1.0	23.2	26.7
% Head Length						
Head-eye Length (5–7)	32.7	20	34.7	2.6	30.5	39.0
Orbit Diameter (4–5)	20.8	20	22.6	1.4	20.4	25.1
Snout Length (1–4)	61.2	20	57.6	2.0	54.5	61.7
Internares Width (2–3)	14.8	20	15.5	2.0	11.2	19.4
Interorbital Width (5–6)	51.1	20	50.5	3.1	43.7	56.2
Head Depth (7–12)	67.8	20	69.0	1.7	65.7	72.2
Mouth Length (1–24)	46.2	20	45.8	2.0	41.0	48.4
Mouth Width (21–22)	53.1	20	50.3	3.0	43.8	57.4
Barbel Length (22–23)	11.0	20	11.3	1.8	7.9	14.0
Dentary Tooth Cup Length (25–26)	20.0	20	19.1	1.8	15.1	23.0
Premaxillary Tooth Cup Length (27–28)	19.2	20	19.3	1.7	16.4	22.6

Eye moderately sized, dorsal rim of orbit forming tall crest that continues forward to area just anterior of nares as low, rounded ridge. Iris operculum present. Interorbital space largely flat, but with slight, rounded, median hump that is contiguous with rounded ridge on snout formed from mesethmoid. Parieto-supraoccipital pointed posteriorly with the posterior point raised above nuchal region in small crest. Infraorbitals, frontal, nasal, compound pterotic, and parieto-supraoccipital supporting odontodes. Preopercle not supporting odontodes. Opercle generally covered by plates and not supporting odontodes although one to four may be present, particularly in smaller individuals.

Lips covered with short, wide papillae. Lower lip wide, upper lip narrow. Edge of lower lip with small crenulae. Maxillary barbel only barbel present, reaching about one third of distance to gill opening.

Median plates 25–26 (mode 26). Plates unkeeled, but first four or five plates of mid-ventral series bent to form slight ridge. Five caudal peduncle plate rows. Plates on all dorsolateral surfaces of body except for extreme edge of snout that only has a narrow column of platelets on either side of the snout tip. Throat mostly covered in platelets except for area right below lower lip. Abdomen covered in platelets except for broad region just anterior to level of pelvic-fin spine insertions, laterally below pelvic girdle, and small region around anus. Evertible cheek plates supporting hypertrophied odontodes that can be everted perpendicular to head. Cheek odontodes 18–40 (mode 32). Longest evertible cheek odontode reaching to about level of posterior edge of pectoral-fin spine. Hypertrophied cheek odontodes relatively weak. Odontodes slightly longer than average body odontodes present along dorsal-, adipose-, pelvic-, caudal-, and pectoral-fin spines; larger individuals with hypertrophied odontodes at tip of pectoral spine.

Dorsal fin ii,7; dorsal spinelet *V*-shaped, dorsal-fin locking mechanism present, last ray of dorsal fin not reaching preadipose plate when adpressed. Adipose fin with single preadipose plate and moderately long spine. Caudal fin i,14,i; caudal fin forked, ventral lobe longer than dorsal lobe; dorsal and ventral procurrent caudal rays five. Pectoral fin i,6; pectoral-fin spine reaching just posterior to pelvic fin when adpressed ventral to pelvic fin. Pelvic fin i,5; pelvic-fin spine extending to posterior end of base of anal fin when adpressed. Anal fin i,4; anal-fin spine slightly shorter than first ray.

Teeth bicuspid with lateral lobe three-quarters length of medial lobe and distal tip of lateral cusp one-half width of tip of medial cusp. 39–72 left dentary teeth (mode 56). 41–73 left premaxillary teeth (mode 64).

#### Color.

Base color red brown, intensity of red greater in smaller specimens. Head and nape almost completely dark brown with some extremely small spots faintly visible on posterodorsal surface of head and nape, many of the spots combining to form vermiculations. Compound pterotic slightly lighter than rest of head and small spots slightly more evident. Pectoral fin dark brown with faint, large, oblong spots along leading edge. Pelvic fin as pectoral but lighter. Dorsal fin with oblong spots along spine, rays red brown, and membranes dark. Caudal fin with three to five bands that may be regular (contiguous along height of fin) or irregular (ventral and dorsal parts offset); lighter interspaces red brown, usually slightly narrower than dark bands (the largest individual examined has the light interspaces much narrower than the bands, which are very irregular). Body with four saddles, first below middle of dorsal fin, second with anterior half below posterior end of dorsal fin and posterior half behind dorsal fin, third beginning one to two plates anterior of preadipose plate to about posterior third of adipose-fin membrane, and fourth beginning just posterior to adipose fin to end of caudal peduncle; first and second saddles and usually third connected at median plate series; saddles appear to be formed of two bars each that fuse as specimens get older, and connection between bands form because the ventral sides appear to get darker with age. Ventral surface uniformly light except for the present of blotches from anterior insertion of anal fin to caudal fin, which may or may not be extensions of the saddles onto the ventral surface.

#### Sexual dimorphism.

It appears that some of the larger specimens (presumably male) are slightly more hispid, suggesting that nuptial males may develop hypertrophied odontodes on the lateral plates; however, no specimens have hypertrophied odontodes. The larger specimens also have the odontodes on the pectoral-fin spines moderately hypertrophied, which may also be a nuptial male characteristic.

#### Distribution.

Known only from the type locality in the rio Madeira drainage of Brazil (Fig. 3).

#### Etymology.

*Ephippiata* is Latin for saddled and refers to the presence of saddles in this species.

#### Remarks.

**Figure 2.**
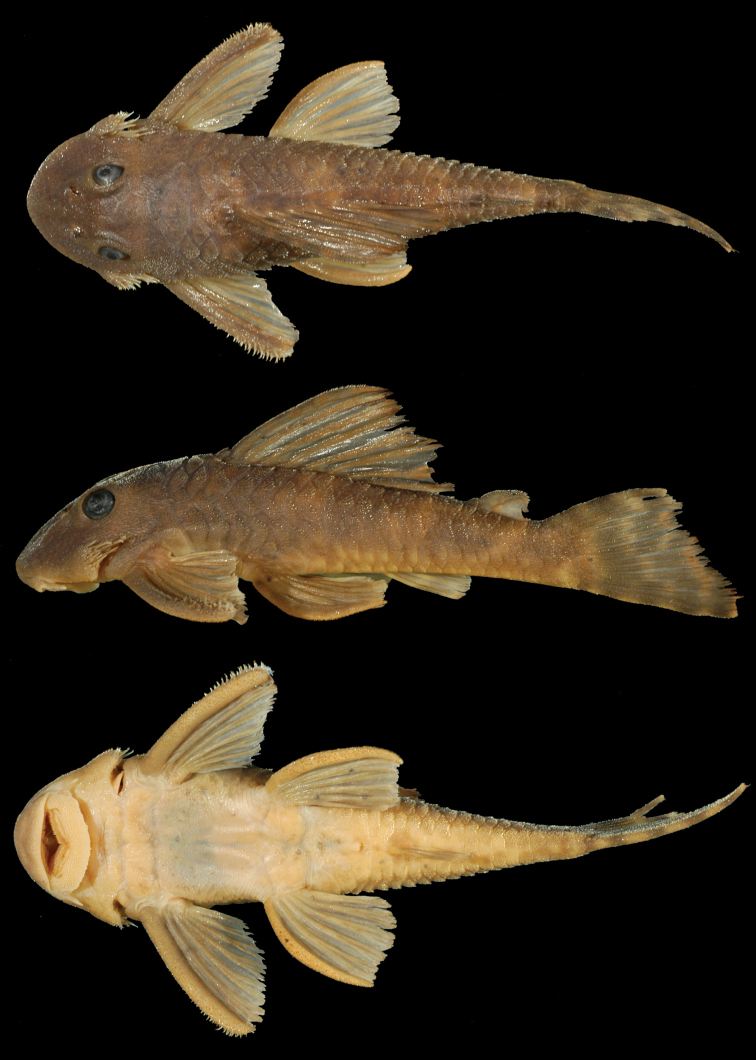
Dorsal, lateral, and ventral views of holotype of *Peckoltia ephippiata* sp. n., MCP 35627, 101.7 mm SL. Photos by J. W. Armbruster.

Many of the specimens in the type series contain a significant load of larval *Neascus*-type metacercariae (visible as black spots on the body and fins in Figure 2). This type of trematode burrows in the skin as larvae and the host mounts a response whereby pigment cells surround the cyst, making the cysts black (C. Sunderman and K. Hayden, pers. comm.).

### Peckoltia greedoi

Taxon classificationAnimaliaSiluriformesLoricariidae

Armbruster, Werneke & Tan sp. n.

http://zoobank.org/F5A921BA-2746-44D2-9D01-E29EBA6A43DF

Fig. 4 , Table 3

#### Type locality.

Brazil, South America

#### Holotype.

MCP 21972, 78.0 mm SL, BRAZIL, Pará, río Gurupi on BR 316 at border of Pará and Maranhão, -01.8003°, -046.3167°, 23 Jul 1998, R. Reis, J. P. Silva, E. Pereira, J. Montoya.

#### Paratypes.

ANSP 197617, 2, 56.0–67.4, AUM 65117, 2, 57.6–71.9, MCP 48396, 23, 46.3–75.8, MNRJ 42663, 2, 55.2–71.5, same locality data as holotype.

#### Diagnosis.

*Peckoltia greedoi* can be separated from *Peckoltia pankimpuju* by having well developed color and eyes; from all other *Peckoltia* except *Peckoltia ephippiata* by having the head uniformly colored (vs. large spots, mottling, short lines, or thick dark areas; faint spots are present in *Peckoltia ephippiata*, but are not obvious); from all *Peckoltia* except *Peckoltia braueri*, *Peckoltia capitulata*, *Peckoltia compta*, *Peckoltia lujani*, *Peckoltia oligospila*, *Peckoltia otali*, and *Peckoltia stimulata* by having the abdomen largely naked posterior to the pectoral girdle (vs. only small naked patches at insertions of pelvic fins); from all *Peckoltia* except *Peckoltia ephippiata*, *Peckoltia furcata*, *Peckoltia lujani*, *Peckoltia pankimpuju*, and *Peckoltia sabaji* by having the dentaries meet at an angle greater than 90°; from *Peckoltia ephippiata* by having fewer teeth (*Peckoltia greedoi*: 16–39 dentary, 20–38 premaxillary; *Peckoltia ephippiata*: 39–72 dentary, 41–73 premaxilary), by having faint spots forming bands in the dorsal fin, and by having platelets maximally present below pectoral girdle and in a narrow, lateral column just posterior to pectoral fin, and below pelvic girdle (vs. platelets on the central region of the abdomen posterior to the pectoral girdle present); and by lacking slight keels on the lateral plates (vs. keels present, strongest on median series); from *Peckoltia lujani* by having no spots on the posterodorsal surface of head and nape (vs. large spots), and by having the pectoral-fin spine relaxed position angled dorsally, pointing at insertion of dorsal fin (vs. pectoral-fin spine angled only slightly dorsally, pointing maximally to dorsal insertion of caudal fin) and pectoral-fin spine reaching two or more plates of the ventral series beyond the pelvic base when adpressed ventral to pelvic fin (vs. less than one plate).

*Peckoltia greedoi* differs from *Etsaputu* by having greater than six evertible cheek odontodes, the largest of which extends posterior to the eye (vs. six or fewer, the largest not extending beyond the exposed portion of the opercle). *Peckoltia greedoi* can be separated from *Hemiancistrus* (except ‘Hemiancistrus’ landoni) and *Ancistomus* by having prominent dorsal saddles (vs. dark or light spots or entirely dark); and from all *Hemiancistrus* and *Ancistomus* by having bands in the caudal fin and no free spots (vs. bands absent or present with some free spots) and bands in the dorsal fin (vs. spots or no markings). *Peckoltia greedoi* can be separated from *Peckoltichthys bachi* by having no spots on the head (vs. large dark spots or mottling); by having the eyes high on the head with the dorsal rim of the orbit higher than the interorbital space (vs. low on the head, dorsal rim of orbit lower than interorbital space), and by having small plates on the abdomen (vs. relatively large).

*Peckoltia greedoi* is very similar to *Peckoltia vittata*. It differs from *Peckoltia vittata* by having the dentaries meeting at an angle >90° (vs. <90°), by having a shallower slope of the head (~30° from snout tip to orbit, vs. >45°), no change in slope of head from anterior margin of orbit to tip of parieto-supraoccipital (vs. angle becoming much shallower beyond orbits), head appearing narrower and longer when placed side-by-side with similar size specimens, abdomen without platelets between pectoral and pelvic girdles (vs. platelets present), pectoral-fin spine reaching two or more plates beyond pelvic-fin base when adpressed ventral to pelvic fin (vs. less than one plate).

#### Description.

Morphometrics in Table 3. Counts and measurements based on 30 specimens. Small to medium-sized loricariids, largest specimen examined 78.0 mm SL. Body moderately elongate. Head and nape forming arc from tip of snout to insertion of dorsal fin. Dorsal slope decreasing in straight line to insertion of dorsal procurrent caudal rays then ascending to caudal fin. Body depth greatest below insertion of dorsal fin. Ventral profile flat to caudal fin. Caudal peduncle triangular in cross section with dorsal surface flattened. Body widest at insertion of pectoral fins, narrowest at insertion of caudal fin. Snout rounded.

**Table 3.** Selected morphometrics of *Peckoltia greedoi*. Numbers in parentheses refer to landmark numbers in Armbruster (2003).

	Holotype	N	Mean	SD	Min	Max
SL, mm (1–20)	77.8	30			45.3	78.0
%SL						
Predorsal Length (1–10)	44.7	30	44.2	1.2	42.1	47.1
Head Length (1–7)	35.8	30	36.8	1.0	35.0	38.8
Head–dorsal Length (7–10)	8.5	30	7.5	0.7	6.0	8.8
Cleithral Width (8–9)	28.2	30	28.7	1.0	26.8	30.7
Head-pectoral Length (1–12)	27.1	30	26.8	1.1	24.5	29.5
Thorax Length (12–13)	20.5	30	21.9	1.3	19.7	25.2
Pectoral-spine Length (12–29)	32.5	30	31.9	1.2	29.7	33.8
Abdominal Length (13–14)	23.3	30	22.8	1.1	20.2	26.1
Pelvic-spine Length (13–30)	27.7	30	27.1	1.6	23.0	31.7
Postanal Length (14–15)	35.3	30	34.2	1.1	31.7	35.9
Anal-fin spine Length (14–31)	16	30	14.8	1.0	12.4	16.4
Dorsal–pectoral Distance (10–12)	27.9	30	28.5	1.1	26.4	30.6
Dorsal spine Length (10–11)	broken	25	31.3	2.5	26.4	36.4
Dorsal-pelvic Distance (10–13)	24.3	30	23.0	1.0	20.9	25.1
Dorsal-fin base Length (10–16)	26.3	30	25.8	1.3	24.0	30.4
Dorsal-adipose Distance (16–17)	16.3	30	16.0	1.2	12.4	18.2
Adipose-spine Length (17–18)	9.8	30	10.0	1.1	8.2	13.2
Adipose-upper caudal Distance (17–19)	16.5	30	16.0	1.5	13.9	21.0
Caudal-peduncle Depth (15–19)	11.1	29	12.3	0.7	11.1	14.3
Adipose-lower caudal Distance (15–17)	23.4	29	22.8	1.5	19.7	26.4
Adipose-anal Distance (14–17)	19	30	18.4	1.2	16.6	21.3
Dorsal-anal Distance (14–16)	15.9	30	15.5	0.5	14.7	16.5
Pelvic-dorsal Distance (13–16)	25.2	30	25.9	1.4	23.6	30.6
% Head Length						
Head-eye Length (5–7)	36.7	30	35.2	1.4	32.2	37.7
Orbit Diameter (4–5)	22.6	30	22.9	1.4	18.0	25.4
Snout Length (1–4)	60.6	30	58.1	1.5	54.9	60.9
Internares Width (2–3)	12.9	30	13.3	0.7	11.7	14.3
Interorbital Width (5–6)	48.9	30	46.1	1.7	42.7	49.8
Head Depth (7–12)	69.6	30	67.4	1.5	64.7	70.4
Mouth Length (1–24)	50.3	30	46.3	3.1	37.7	53.4
Mouth Width (21–22)	51.9	27	47.9	3.6	41.1	54.5
Barbel Length (22–23)	14.9	29	15.1	2.4	12.2	23.7
Dentary Tooth Cup Length (25–26)	15.3	30	18.2	2.1	14.7	22.4
Premaxillary Tooth Cup Length (27–28)	17.5	30	17.0	1.2	14.9	20.6

Eye moderately sized, dorsal rim of orbit forming tall crest that continues forward to area just anterior of nares as low, rounded ridge. Iris operculum present. Interorbital space flat anteriorly, but with slight, rounded, median hump posteriorly that is contiguous with ridge of parieto-supraoccipital. Parieto-supraoccipital pointed posteriorly with the posterior point raised above nuchal region in small crest. Infraorbitals, frontal, nasal, compound pterotic, and parieto-supraoccipital supporting odontodes. Preopercle not supporting odontodes. Opercle generally covered by plates and not supporting odontodes although one to four may be present, particularly in smaller individuals.

Lips covered with short, wide papillae. Lower lip wide, upper lip narrow. Edge of lower lip with small crenulae. Maxillary barbel only barbel present, reaching about one third of distance to gill opening.

Median plates 24–26 (mode 25). Plates unkeeled, but first four or five plates of mid-ventral series bent to form slight ridge. Five caudal peduncle plate rows. Plates on all dorsolateral surfaces of body except for extreme edge of snout that only has a narrow column of platelets on either side of the snout tip. Throat mostly naked with platelets confined to lateral margins. Pectoral girdle covered in platelets on ventral surface. Breast naked except for one or two platelets laterally between pectoral and pelvic fin insertions. Abdomen covered in platelets behind last pelvic-fin ray insertion except for lateral margins and small region around anus. Evertible cheek plates supporting hypertrophied odontodes that can be everted perpendicular to head. Cheek odontodes 17–40 (mode 33). Longest evertible cheek odontode reaching to about level of posterior edge of pectoral-fin spine. Hypertrophied cheek odontodes relatively weak. Odontodes slightly longer than average body odontodes present along dorsal-, adipose-, pelvic-, caudal-, and pectoral-fin spines; larger individuals with hypertrophied odontodes at tip of pectoral spine.

Dorsal fin ii,7; dorsal spinelet *V*-shaped, dorsal-fin locking mechanism present, last ray of dorsal fin not reaching preadipose plate when adpressed. Adipose fin with single preadipose plate and moderately long spine. Caudal fin i,14,i; caudal fin forked, ventral lobe longer than dorsal lobe; dorsal and ventral procurrent caudal rays five. Pectoral fin i,6; pectoral-fin spine reaching just posterior to pelvic fin when adpressed ventral to pelvic fin. Pelvic fin i,5; pelvic-fin spine extending to posterior end of base of anal fin when adpressed. Anal fin i,4; anal-fin spine slightly shorter than first ray.

Teeth bicuspid with lateral lobe one-half to three-quarters length of medial lobe and distal tip of lateral cusp one-half width of tip of medial cusp. 16–39 left dentary teeth (mode 28). 20–38 left premaxillary teeth (mode 27).

#### Color.

Base color red brown. Head and nape almost completely dark brown. Pectoral-fin spine dark brown with faint, large, oblong spots on dorsal surface forming faint bands across pectoral-fin rays. Pelvic fin as pectoral but lighter. Dorsal fin with oblong spots along spine forming bands across dorsal-fin rays. Caudal fin with three to four bands that may be regular (contiguous along height of fin) or irregular (ventral and dorsal parts offset); lighter interspaces tan, usually equal in diameter to dark. Body with three saddles, first below middle of dorsal fin, second with anterior half below posterior end of dorsal fin and posterior half behind dorsal fin, and third beginning at preadipose plate to about middle adipose-fin membrane; saddles connected at median plate series; saddles appear to be formed of two bars each that fuse as specimens get older, and connection between bands form because the ventral sides appear to get darker with age. Ventral surface uniformly light except for the present of blotches from anterior insertion of anal fin to caudal fin, which may or may not be extensions of the saddles onto the ventral surface.

#### Sexual dimorphism.

None observed.

#### Distribution.

**Figure 3.**
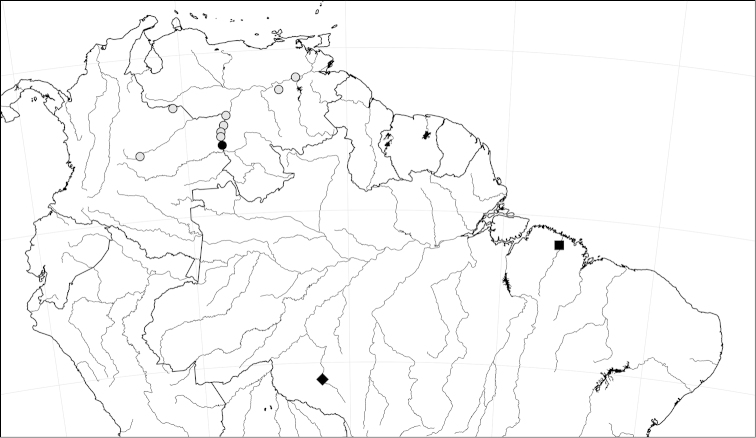
Distribution of the new species of *Peckoltia*: *Peckoltia ephippiata* (diamond), *Peckoltia greedoi* (square), and *Peckoltia lujani* (dots, black dot is type locality).

Known only from the type locality in the rio Gurupi drainage of Brazil (Fig. 3).

#### Remarks.

Armbruster (2008) reported *Peckoltia vittata* from the rio Gurupi drainage; however, this was based on the collection identified here as *Peckoltia greedoi*. Characters to separate *Peckoltia greedoi* from *Peckoltia vittata* are detailed in the diagnosis.

#### Etymology.

Named for Greedo of Rodia, a bounty hunter killed by Han Solo in Chalmun’s Spaceport Cantina in the movie “Star Wars: Episode IV – A New Hope” (Lucasfilm, Twentieth Century Fox, 1977) with whom this species shares a remarkable resemblance.

**Figure 4.**
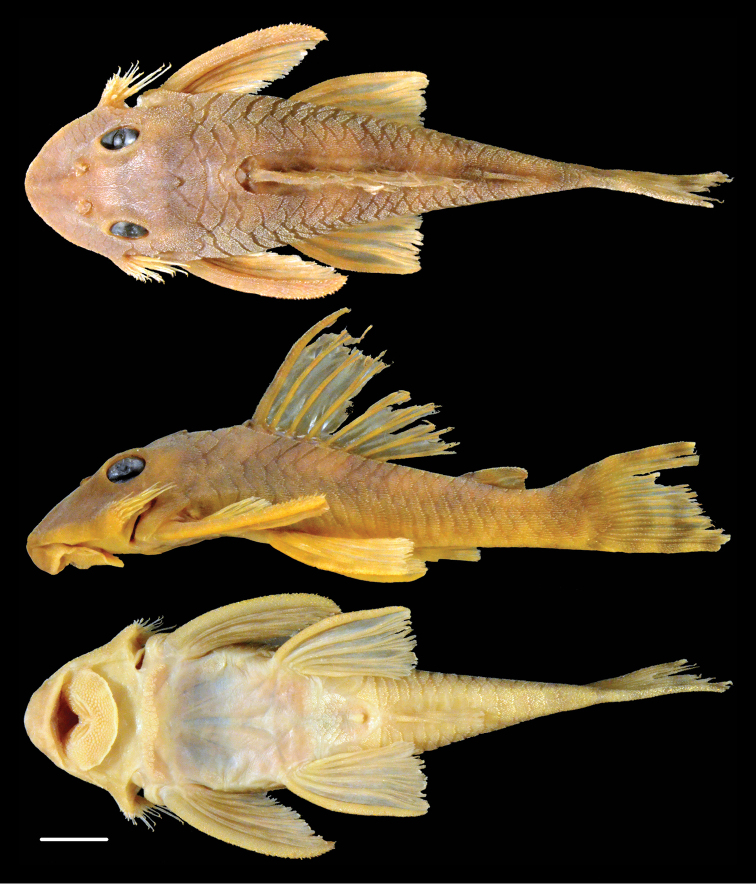
Dorsal, lateral, and ventral views of holotype of *Peckoltia greedoi* sp. n., MCP 21972, 77.8 mm SL. Photos by J. W. Armbruster.

### Peckoltia lujani

Taxon classificationAnimaliaSiluriformesLoricariidae

Armbruster, Werneke & Tan sp. n.

http://zoobank.org/2AAE8CC5-5B6A-4A50-A972-777D4FB74EAF

Fig. 5 , Table 4

#### Type locality.

Venezuela, South America

#### Holotype.

AUM 53523, 75.1 mm SL, VENEZUELA, Amazonas, río Orinoco at Paso Ganado, 38 km NNW of San Fernando de Atabapo, 04.3842°, -067.7747°, 27 Mar 2010, N.K. Lujan, D.C. Werneke, M.H. Sabaj, T. Carvalho, V. Meza, and O. León.

#### Paratypes.

ANSP 162174, 13, 46.0–74.3, VENEZUELA, Amazonas, río Orinoco at El Burro, 06.2°, -067.4333°, 26 Nov 1985, B. Chernoff et al.; AUM 43008, 4 nm, 19.8–32.4, VENEZUELA, Amazonas, río Orinoco dr., río Orinoco, at Puerto Venado 2 km NW of Samariapo and 56.4 km SSW of Puerto Ayacucho, 05.2106°, -067.8049°, 26 Feb 2005, N.K. Lujan, D.C. Werneke, M.H. Sabaj, M. Arce, R. Betancur, and T.E. Wesley; AUM 53474, 1, 37.4, VENEZUELA, Amazonas, rio Orinoco at Raudales Atures, 8.3 km SSW of Puerto Ayacucho, 05.5989°, -067.6139°, 23 Mar 2010, N.K. Lujan, D.C. Werneke, M.H. Sabaj, T. Carvalho, V. Meza; AUM 53979, 2, 31.6–34.3, VENEZUELA, Amazonas, rio Orinoco at Merey, 97.6 km N of San Fernando de Atabapo, 04.9178°, -067.8329°, 18 Apr 2010, J. Birindelli, N.K. Lujan, and V. Meza; MCNG 56579, 1,62.9, MCP 48401, 1, 57.8, same data as holotype; ROM 93352, 12, 38.0–64.5, VENEZUELA, Amazonas, rio Orinoco across channel from Puerto Venado (near Samariapo), 56.7 km south-southwest of Puerto Ayacucho, 05.2095°, -067.8095°, 24 Mar 2010, N.K. Lujan, M.H. Sabaj, D.C. Werneke, V. Meza, and T. Carvalho.

#### Other material.

ANSP 166770, 1, 61.3, VENEZUELA, Bolivar, río Orinoco dr., rio Aro, Salto El Pajaro, 18 Oct 1987, M. Rodriguez; ICNMHN 1480, 13, 35.3–76.3 (4 nm), COLOMBIA, Meta, río Meta - río Orinoco dr., río Negro on the Villavicencio - Puerto Lopez road, (4.1025°, -072.9368°), 11 Jan 1988, H. Silvergrip; ICNMH 9096, 1, 79.5, COLOMBIA, Arauca, río Meta - río Orinoco dr., Caño Ormedillo, Arauca-Caño Norte road, (06.8514°, -070.6486°) 27 Feb 1977, P. Cala; MCNG 19318, 2 nm., VENEZUELA, Bolivar, río Orinoco to the east of Ciudad Bolivar in the population of El Rosario, 08.3167°, -063.0833°, 24 Sep 1987, G. Feo, R. Morales, and H. Barbarino.

#### Diagnosis.

*Peckoltia lujani* can be separated from *Peckoltia pankimpuju* by having well developed color and eyes; from all *Peckoltia* except *Peckoltia braueri*, *Peckoltia capitulata*, *Peckoltia compta*, *Peckoltia greedoi*, *Peckoltia oligospila*, *Peckoltia otali*, and *Peckoltia stimulata* by having the abdomen largely naked posterior to the pectoral girdle (vs. only small naked patches at insertions of pelvic fins); from all *Peckoltia* except *Peckoltia furcata*, *Peckoltia greedoi*, *Peckoltia lujani*, *Peckoltia pankimpuju*, and *Peckoltia sabaji* by having the dentaries meet at an angle greater than 90°; from *Peckoltia ephippiata* and *Peckoltia greedoi* by having large spots or blotches on the posterolateral surface of head and nape (vs. very small, very faint spots); from *Peckoltia ephippiata* and by lacking slight keels on the lateral plates, particularly the median series (vs. slight keels present), by having bands in the dorsal fin (vs. dorsal fin with light rays and dark membranes), by having fewer teeth (*Peckoltia ephippiata*: 39–72 dentary, 41–73 premaxillary; *Peckoltia lujani*: 20–37 dentary, 23–45 premaxillary); from *Peckoltia greedoi* by having the pectoral-fin spine relaxed position only slightly dorsally, pointing maximally to dorsal insertion of caudal fin (vs. angled dorsally, pointing at insertion of dorsal fin) and pectoral-fin spine reaching less than one plate of the ventral series beyond the pelvic base when adpressed ventral to pelvic fin (vs. two or more).

*Peckoltia lujani* differs from *Etsaputu* by having greater than six evertible cheek odontodes, the largest of which extends posterior to the eye (vs. six or fewer, the largest not extending beyond the exposed portion of the opercle). *Peckoltia lujani* can be separated from *Hemiancistrus* (except ‘Hemiancistrus’ landoni) and *Ancistomus* by having prominent dorsal saddles (vs. dark or light spots or entirely dark); and from all *Hemiancistrus* and *Ancistomus* by having bands in the caudal fin and no free spots (vs. bands absent or present with some free spots) and bands in the dorsal fin (vs. spots or no markings). *Peckoltia ephippiata* can be separated from *Peckoltichthys bachi* by having no spots on the head (vs. large dark spots or mottling); by having the eyes high on the head with the dorsal rim of the orbit higher than the interorbital space (vs. low on the head, dorsal rim of orbit lower than interorbital space), and by having small plates on the abdomen (vs. relatively large).

*Peckoltia caenosa* is known from the same region as *Peckoltia lujani* and can be difficult to tell apart when juveniles. *Peckoltia lujani* differs from adult *Peckoltia caenosa* by lacking vermiculations on the abdomen and head, and from all *Peckoltia caenosa* by having the dentaries meet in a broad arc that is greater than 120° (vs. meeting at an angle less than 90°), and by having fewer teeth (all except one specimen with 24–37 dentary teeth and 22–45 premaxillary teeth [16 and 19 respectively in aberrant specimen] vs. 10–18 dentary teeth and 11–21 premaxillary teeth).

#### Description.

Morphometrics in Table 4, counts and measurements based on 25 specimens unless noted. Largest specimen examined 75.1 mm SL. Body moderately elongate. Head and nape forming arc from tip of snout to anterior of parieto-supraoccipital, rising more rapidly to parieto-supraoccipital crest, and then more slowly to dorsal-fin. Dorsal slope decreasing in straight line to insertion of dorsal procurrent caudal rays then ascending to caudal fin. Body depth greatest below insertion of dorsal fin. Ventral profile flat to ventral procurrent caudal-fin rays, and then sloped ventrally. Caudal peduncle oval in cross section with dorsal and ventral surfaces flattened. Body widest at insertion of pectoral fins, narrowest at insertion of caudal fin. Snout rounded.

**Table 4.** Selected morphometrics of *Peckoltia lujani*. Numbers in parentheses refer to landmark numbers in Armbruster (2003).

	Holotype	N	Mean	SD	Max	Min
SL, mm (1–20)	75.1	34			31.6	75.1
%SL						
Predorsal Length (1–10)	41.2	34	42.3	1.2	39.4	44.3
Head Length (1–7)	33.5	34	35.6	1.5	32.9	41.2
Head–dorsal Length (7–10)	9.3	34	7.1	1.0	5.3	9.5
Cleithral Width (8–9)	25.5	34	27.8	1.4	23.1	29.6
Head-pectoral Length (1–12)	27.9	34	26.7	1.5	22.9	29.7
Thorax Length (12–13)	21.5	34	23.7	1.7	21.3	30.7
Pectoral-spine Length (12–29)	27.7	34	28.7	1.0	26.6	30.9
Abdominal Length (13–14)	22.7	34	22.1	0.9	19.5	23.7
Pelvic-spine Length (13–30)	25.4	34	25.0	1.4	22.5	28.6
Postanal Length (14–15)	35.9	34	33.6	1.5	29.4	35.9
Anal-fin spine Length (14–31)	14.3	34	12.9	1.0	10.1	15.3
Dorsal–pectoral Distance (10–12)	25.0	31	26.7	1.2	24.3	29.6
Dorsal spine Length (10–11)	26.8	32	26.4	2.7	17.6	31.4
Dorsal-pelvic Distance (10–13)	19.5	34	21.0	1.7	15.5	23.6
Dorsal-fin base Length (10–16)	28.2	34	26.7	1.4	23.2	29.9
Dorsal-adipose Distance (16–17)	14.4	34	14.3	2.0	9.7	18.6
Adipose-spine Length (17–18)	7.9	34	9.3	1.6	6.6	12.7
Adipose-upper caudal Distance (17–19)	15.9	34	16.9	2.3	12.3	21.2
Caudal-peduncle Depth (15–19)	9.2	34	9.8	1.0	8.1	12.1
Adipose-lower caudal Distance (15–17)	21.5	34	22.1	1.9	18.9	26.9
Adipose-anal Distance (14–17)	18.0	34	17.9	1.8	14.7	22.4
Dorsal-anal Distance (14–16)	12.6	34	13.4	0.9	11.4	15.0
Pelvic-dorsal Distance (13–16)	24.0	34	24.5	1.7	21.3	27.8
% Head Length						
Head-eye Length (5–7)	35.6	34	37.4	2.9	29.0	45.8
Orbit Diameter (4–5)	20.4	34	20.7	2.1	16.6	24.1
Snout Length (1–4)	55.2	34	55.7	3.0	45.0	61.6
Internares Width (2–3)	15.0	33	14.8	1.9	11.4	19.5
Interorbital Width (5–6)	44.8	43	43.7	3.8	36.5	54.8
Head Depth (7–12)	63.3	43	64.8	3.4	51.5	73.2
Mouth Length (1–24)	53.1	44	49.6	3.4	42.4	57.1
Mouth Width (21–22)	55.6	44	50.1	5.1	41.6	58.8
Barbel Length (22–23)	15.8	42	13.8	2.3	10.2	19.7
Dentary Tooth Cup Length (25–26)	15.5	40	13.0	2.0	10.1	17.9
Premaxillary Tooth Cup Length (27–28)	16.5	42	13.9	1.5	10.8	17.0

Eye moderately sized, dorsal rim of orbit forming moderate crest that continues forward of orbit to area just anterior of nares as a low, rounded ridge. Iris operculum present. Interorbital space flat. Parieto-supraoccipital pointed posteriorly with a moderate crest formed along near entire length of parieto-supraoccipital. Infraorbitals, frontal, nasal, compound pterotic, and parieto-supraoccipital supporting odontodes. Preopercle not supporting odontodes. Opercle generally covered by plates and not supporting odontodes although one to two may be present, particularly in smaller individuals.

Lips covered with short, wide papillae. Lower lip wide, upper lip narrow. Edge of lower lip with small crenulae. Maxillary barbel only barbel present, reaching about one third of distance to gill opening.

Median plates 25–27 (mode 26). Plates unkeeled, but first four or five plates of mid-ventral series very slightly bent to form slight ridge. Five caudal peduncle plate rows. Plates on all dorsolateral surfaces of body except for oval naked area at snout tip. Throat with a few plates posterior to lip. Abdomen only with few sparse platelets below pectoral girdle and in narrow column posterolaterally to pectoral girdle in type series (some platelets below pelvic girdle in Meta specimens). Evertible cheek plates supporting hypertrophied odontodes that can be everted perpendicular to head. Cheek odontodes 5–49 (mode 25). Longest evertible cheek odontode reaching to about level of posterior edge of pectoral-fin spine. Hypertrophied cheek odontodes relatively weak. Odontodes slightly longer than average body odontodes present along dorsal-, adipose-, pelvic-, caudal-, and pectoral-fin spines; larger individuals with hypertrophied odontodes at tip of pectoral spine.

Dorsal fin ii,7; dorsal spinelet *V*-shaped, dorsal-fin locking mechanism present, last ray of dorsal fin not reaching preadipose plate when adpressed. Adipose fin with single preadipose plate and moderately long spine. Caudal fin i,14,i; caudal fin forked, ventral lobe longer than dorsal lobe; dorsal procurrent caudal rays five, and ventral procurrent caudal rays four to five (mode five; n=24 for dorsals). Pectoral fin i,6; pectoral-fin spine reaching just posterior to pelvic fin when adpressed ventral to pelvic fin. Pelvic fin i,5; pelvic-fin spine extending to posterior end of base of anal fin when adpressed. Anal fin i,4; anal-fin spine slightly shorter than first ray.

Teeth bicuspid with lateral lobe three-quarters length of medial lobe and distal tip of lateral cusp one-half width of tip of medial cusp. 20–37 left dentary teeth (mode 32). 23–45 left premaxillary teeth (mode 39).

#### Color.

Base color light tan with brown to black markings. Four dorsal saddles on the body, the first below the middle rays of the dorsal fin, the second below the posterior rays of the dorsal fin and slightly posterior, the third below the adipose fin and slightly anterior, and the fourth at the end of the caudal peduncle. Third and fourth saddles may have anterior extensions or have an anterior projection making them *h*-shaped or may be split nearly in half. Saddles two to four extend to median plate row, saddle one continues to insertion of pectoral fin. Very large blotches or spots present from saddle one to caudal fin. All fins except dorsal always with dark bands with dark areas from about as wide to about twice as wide as light areas. Number of bands increases with size. Dorsal fin coloration complex, ranging from a mix of dark and light spots that may or may not combine to form bands (bands always forming distally). The dark or light spots/bands in dorsal fin may or may not combine with those dorsal or ventral. Dark spots/bands typically darker on the rays distally and on the membranes proximally. Dark spot present between dorsal-fin spinelet and spine. Abdomen with medium spots. Lower surface of caudal peduncle with dark blotches. Juveniles colored as adults, but with bar two extending to insertion of pelvic fin, without anterior extensions of the third and fourth dorsal bars, third and fourth bars extending across ventral margin of caudal peduncle, without spots on the abdomen, and with the spots or blotches on the sides (if present) just between first and second bars.

#### Sexual dimorphism.

None observed.

#### Distribution.

Known from the Meta Drainage near Villavicencio and the Orinoco from the mouth of the Meta to around Ciudad Bolivar (Fig. 3).

#### Remarks.

The type locality was restricted to collection localities in Amazonas, Venezuela. There is variation within the species, and it is possible that other forms may be present within the species. The Colombian specimens (ICNMHN 1480 and ICNMH 9096) differed in shape from the other members of the species, and we do not have the specimens to check their identity, so they were excluded from counts and measurements.

#### Etymology.

Named in honor of the former graduate student of JWA, Dr. Nathan Lujan. Dr. Lujan has led expeditions to some of the most remote regions of South America and obtained some of the most important specimens for the study of loricariid systematics specifically as well as South American fish systematics and ecology in general. In the process, he has given JWA more taxonomic work in the last decade than he had thought possible, and he is very thankful. Dr. Lujan also collected the best specimens known of the species.

**Figure 5.**
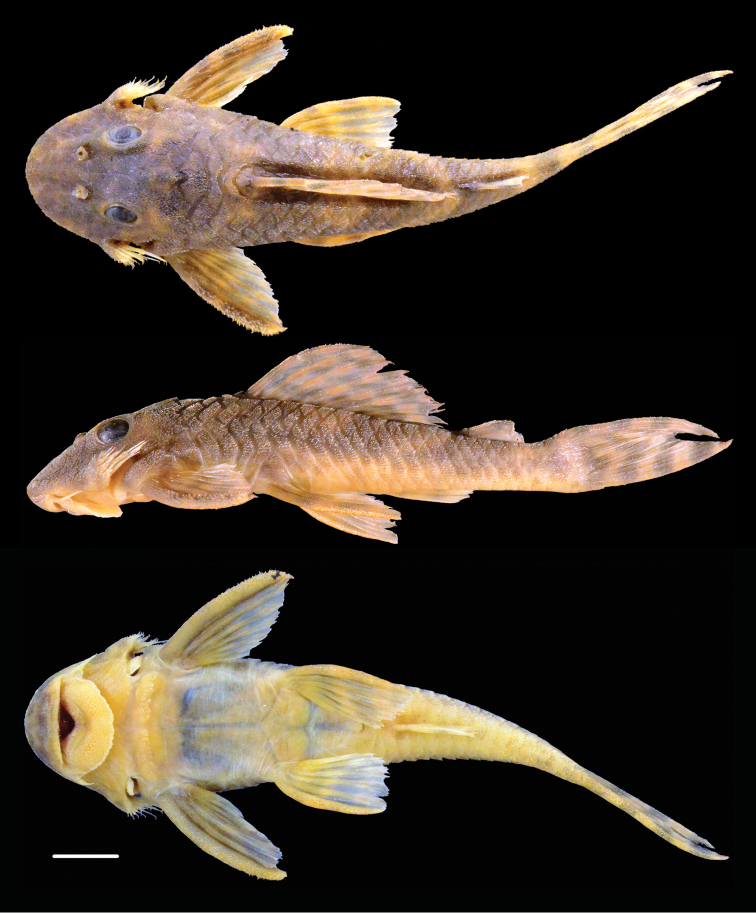
Dorsal, lateral, and ventral views of holotype of *Peckoltia lujani* sp. n., AUM 53523, 75.1 mm SL. Photos by J. W. Armbruster.

## Discussion

We offer a taxonomy for *Hemiancistrus*, *Peckoltia*, and allied genera based mostly on the molecular phylogeny and conclusions of Lujan et al. (2015) (Table 1). We do not currently have characteristics to diagnose the various genera or species groups that arise from this phylogeny, but the molecular phylogeny offers the best case right now for handling these confusing species. The molecular phylogeny suggests that the only species that should be left in *Hemiancistrus* is the type species. *Hemiancistrus medians* is larger than most species that were left in *Hemiancistrus* in Armbruster (2008), has well-developed keels (only present elsewhere in ‘Hemiancistrus’ landoni), and a different body shape. The remainder of the taxa that do not have established genera that they can be placed in will be recognized as species groups in ‘*Hemiancistrus*’ in single quotes until they can be examined further. We recognize three species groups: ‘Hemiancistrus’ chlorostictus group, ‘Hemiancistrus’ guahiborum group, and ‘Hemiancistrus’ landoni group.

*Ancistomus* is recognized as valid with *Peckoltia feldbergae*, *Hemiancistrus micrommatos*, *Hemiancistrus spinosissimus*, and *Hemiancistrus spilomma* along with the type, *Ancistrus snethlageae*. *Ancistomus micrommatos*, *Ancistomus spinosissimus*, and *Ancistomus spilomma* were not examined by Lujan et al. (2015); however, they are very similar in appearance to *Ancistrus snethlageae*, and *Ancistomus spilomma* was sister to *Ancistrus snethlageae* in Armbruster (2008). We cannot find any characteristics to separate *Ancistomus micrommatos*, *Ancistomus spinosissimus*, and *Ancistomus spilomma*, and they may represent the same species.

The *Hemiancistrus annectens* group of Armbruster (1998) shares with *Pterygoplichthys* a connective tissue sheet that connects laterally to the abdominal wall and evertible cheek plates with hypertrophied odontodes. Armbruster (1998, 2004a) suggested that a new genus needed to be described for them, but the species consistently come out as sister to *Hypostomus* in analyses of molecular data (Evans 2002; Lujan et al. 2015), and we recognize the species in *Hypostomus*. Further study will be needed to determine if the group requires its own, separate genus.

The *Hemiancistrus* from southern Brazil and Uruguay are in a polytomy with the *Hemiancistrus annectens* group and the rest of *Hypostomus* in Lujan et al. (2015); however, the group is very different morphologically with all of the characters used to unite the Ancistrini of Armbruster (2004a, 2008). We recognize the group as the ‘Hemiancistrus’ chlorostictus group until its relationships can be examined further. We tentatively consider ‘Hemiancistrus’ cerrado as a member of the ‘Hemiancistrus’ chlorostictus group due to overall similarity in form between it and the southern species.

‘Hemiancistrus’ guahiborum and ‘Hemiancistrus’ subviridis are part of the same clade (though not sister species), are morphometrically very similar (pers. obs.), and we recognize them as the ‘Hemiancistrus’ guahiborum group (Lujan et al. 2015). The species differ greatly in color (mottled brown vs. green with small yellow spots respectively), and represent another example of upper Orinoco loricariids differing strongly in color pattern, but not shape. *Pseudolithoxus* and *Hypancistrus* from the Orinoco have a similar wide variation in color between species (Armbruster and Provenzano 2000; Armbruster et al. 2007; Lujan and Birindelli 2011). ‘Hemiancistrus’ guahiborum and ‘Hemiancistrus’ subviridis are in a clade with *Baryancistrus beggini* and *Baryancistrus demantoides*. The two Orinoco *Baryancistrus* are not closely related to true *Baryancistrus* suggesting that a new genus may need to be described for these four Orinoco species.

We recognize a monotypic ‘Hemiancistrus’ landoni group for the trans-Andean species. The type of ‘Hemiancistrus’ hammarlundi is a juvenile ‘Hemiancistrus’ landoni, and we place it into the synonymy of ‘Hemiancistrus’ landoni.

The three new species of *Peckoltia* differ from *Peckoltia* *sensu stricto* by having straighter jaws (dentaries meeting at an angle greater than 90°). Curiously, the sister to *Peckoltia lujani* in Lujan et al. (2015) is the Orinoco population of *Peckoltia vittata* (likely another undescribed species). The Orinoco *Peckoltia vittata* has the dentaries meet at an angle less than 90° and a deep, short body whereas *Peckoltia lujani* is much flatter and more elongate with straighter jaws.

*Peckoltia sabaji* does belong in *Peckoltia* and is sister to the morphologically similar *Peckoltia furcata*. *Etsaputu relictum* was in a clade with *Peckoltia furcata* + *Peckoltia sabaji* and another undescribed species of *Peckoltia* from the Madeira (Lujan et al. 2015), and was morphometrically similar to *Peckoltia ephippiata*. *Etsaputu* is unusual in that it has very few odontodes on the cheek (six or fewer), and they are not very evertible (Lujan et al. 2011). It was found to be at the base of the Ancistrini in the morphological phylogeny (Armbruster 2008). We feel that more information is needed to determine if *Etsaputu* should be recognized as a synonym of *Peckoltia*.

### Chaetostomus macrops

**Figure 6.**
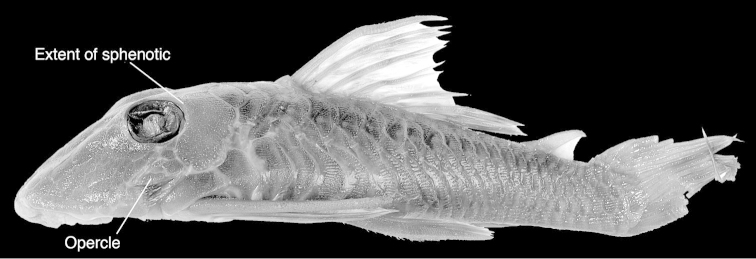
Lateral view of holotype of *Chaetostomus macrops*, ZMUC P30142, 92.7 mm SL. Photos by P. Møller.

*Chaetostomus macrops* Lütken is usually listed as a species of *Hemiancistrus* (Isbrücker 1980, 2001, Fisch-Muller 2003, Ferraris 2007); however, this appears to be incorrect. Photographs from P.R. Møller are excellent (Fig. 6), and indicate that the type is not a *Hemiancistrus*. The type of *Chaetostomus macrops* has odontodes on the opercle over a broad area, a condition that is not seen in *Hemiancistrus*, but which is common to most other ancistrins. In *Hemiancistrus* (as well as *Baryancistrus*, *Hypancistrus*, *Panaque*, *Parancistrus*, and *Peckoltia*) the opercle has its lateral face restricted to a narrow ridge, so if odontodes are present, they are present normally in just a narrow row just one odontode wide. It is also clear on the image that the sphenotic does not have an exterior contact with the last infraorbital; although this feature exhibits some homoplasy, it is a synapomorphy of *Pseudancistrus* (Armbruster 2004b). Also common to *Pseudancistrus*, but not *Hemiancistrus*, *Peckoltia*, or allied genera, is the presence of four to five plates prior to the dorsal fin (vs. three) and a completely naked abdomen (vs. almost always some plates in *Hemiancistrus*).

Cardoso and Lucinda (2003) suggested that *Chaetostomus macrops* is a synonym of *Chaetostomus megacephalus* Günther, which Armbruster (2004a, b) found to be a member of *Pseudancistrus* based on the presence of many synapomorphies (including the lack of exterior contact between the sphenotic and last infraorbital). The type of *Chaetostomus macrops* shares with *Pseudancistrus megacephalus* very large eyes, a wide body, and an unusual color pattern consisting of large white spots on the sides and dark bands on the caudal fin. This color pattern is unusual in loricariids because species with dark bands in the tail usually have dark spots or saddles on the body and those with white spots on the body generally either have spots also on the caudal fin or have the caudal fin entirely dark (*Pseudancistrus brevispinnis* can have a similar color pattern, but it has smaller spots, a narrower body, and smaller eyes). The types of *Chaetostomus macrops* and *Chaetostomus megacephalus* are both likely from Suriname, and the species are synonymous. *Pseudancistrus* *sensu* Armbruster (2004a,b, 2008) was found to be a polyphyletic taxon in Lujan et al. (2015), and we recognize ‘Pseudancistrus’ megacaphalus with its genus in single quotes to indicate it is a taxon that needs further work.

### Hypostomus itacua

*Hypostomus itacua* Valenciennes (supposedly from the La Plata system) is occasionally listed in *Chaetostomus* or *Hemiancistrus* (for example Günther 1864, Isbrücker 1980, Cardoso and Lucinda 2003), but the type appears to be lost. Kner (1854) compared *Ancistrus medians* with *Hypostomus itacua* and stated that both are between his two groups of *Ancistrus*; however, the specimen he referred to as *Ancistrus itacua* is now the type of *Peckoltia braueri*. Weber (2003) indicates that the description in Cuvier and Valenciennes (1840) does not agree with the illustration in Valenciennes (1836), and the 1840 description was used by Günther (1864) to place the species in *Chaetostomus* and Isbrücker (1980) to place it in *Hemiancistrus*. We believe the figure in Valenciennes (1836) is of a *Hypostomus*; however, the species should remain *incertae sedis* in the Loricariidae as in Weber (2003) and Ferraris (2007).

## Supplementary Material

XML Treatment for Peckoltia ephippiata

XML Treatment for Peckoltia greedoi

XML Treatment for Peckoltia lujani

## Supplementary materials

Supplementary material 1 Morphometrics and Meristics for species of *Peckoltia* and *Etsaputu*. Data type: Mesurements and meristics. Explanation note: Morphometirc and meristic data based on Armbruster (2003) for all specimens of *Peckoltia* and *Etsaputu* examined. This dataset is made available under the Open Database License (http://opendatacommons.org/licenses/odbl/1.0/). The Open Database License (ODbL) is a license agreement intended to allow users to freely share, modify, and use this Dataset while maintaining this same freedom for others, provided that the original source and author(s) are credited.Jonathan W. Armbruster, David C. Werneke, Milton Tan

Supplementary material 2 Locality information for all collections of the three species described in this paper. Data type: Occurences. Explanation note: Database containing catalog numbers, numbers of specimens, and localities for the three new species of *Pecoltia* descrived in this paper. This dataset is made available under the Open Database License (http://opendatacommons.org/licenses/odbl/1.0/). The Open Database License (ODbL) is a license agreement intended to allow users to freely share, modify, and use this Dataset while maintaining this same freedom for others, provided that the original source and author(s) are credited.Jonathan W. Armbruster, David C. Werneke, Milton Tan

Supplementary material 3 Morphological characters for *Hemiancistrus medians* Data type: character state data. Explanation note: Character state data based on Armbruster (2004, 2008) for *Hemiancistrus medians*. These data were accidentally removed from Armbruster 2008. This dataset is made available under the Open Database License (http://opendatacommons.org/licenses/odbl/1.0/). The Open Database License (ODbL) is a license agreement intended to allow users to freely share, modify, and use this Dataset while maintaining this same freedom for others, provided that the original source and author(s) are credited.Jonathan W. Armbruster, David C. Werneke, Milton Tan
